# On the Section and Recuperative Property of Tendons

**Published:** 1846-08

**Authors:** 


ART. III.—*On the Section and Recuperative property of Tendons.*—While reading the notice of "Lectures on the Nature and Treatment of Deformities," &c. p. p. 1 and 2 of your Jour nal, (No. for June) my attention was arrested by the four principles laid down by Delpech, and was pleased when it was noted that the second and third of these are in dispute, for surely they should not pass unchallenged. The authorities for, and against are valuable, and were they all on the side of Delpech might constitute a formidable *argumentum ad verecundiam*, sufficient, perhaps, to restrain any *modest* person from the attempt to controvert them; but as one may wish to be in good company, however modest, permit me to speak to the question for a brief space, after having copied the objectionable second and third principles, for the purpose of facilitating reference.

"2d. Immediately after division of the tendon, the divided ends should be brought into contact with each other, and kept in this position by a suitable apparatus during the entire period necessary for their union."

"3d. Inasmuch as it can only take place by an intermediate fibrous substance, this substance, before it has become firm, can, and should be extended gradually and carefully, until it has assumed a degree of length equal to the shortened muscle."

In this immediate vicinity, perhaps, more than any other, is there an under-tone in the profession, (surgical) counter to all section of tendons, and for substitute therefor, it is believed that apparatus and extention are preferable and amply sufficient, where *any* practice is feasible. For what reason, if any, aside from the above, objections are waged against tenotomy I am not informed, and therefore would, through your columns, call attention to the existing fact, in hopes some advocate of apparatus solely, may be induced to give his views upon the subject.

The writer of this has but little experience in operations by section of tendons, but his experience and sanction have always called for the knife. Having practised upon infants, youth of middle age, and the adult, and always, so far as practicable, counter to 'principles' third and fourth of Delpech, I have seen no cause to change that course. I say so far as practicable, for in the adult, the fixity of the joint, by rigidity of ligaments, oppose much separation of the divided tendon, other than that induced by the contraction of the corresponding muscle.

As within the past three years I have yielded to the tone of opinion hereabouts and not operated, (here I may remark, pecuniary reward is seldom equal to the care required) and until these reasons are removed may not operate again, I may as well spare my *modesty* and reply to the second principle. Though I do not doubt the mode of Delpech has also its success, the other mode, not his, has surely this advantage:—It requires less of the *tactus eruditus* in determining the time for the putting stress, in the newly formed fibrous substance. I restrict myself to the following case in answer to the third, and it covers part of the second principle.

Lewis ———, age —, out patient, residence, Buffalo ;—applied in my office for treatment of a gaping, indurated wound on anterior carpal surface. For history, I learn this wound was caused by accidental incision while in use of a pocket knife ; immediately thereafter a physician, not in good standing, dressed it with adhesive plaster solely, about two weeks from date. Motion and other causes had induced separation of the lips of the wound and exposure of the common flexor tendons of the fingers. Advised cataplasms. Purposed for future; approximation and retention by suture and adhesive straps, and confinement of the wrist by splint, to prevent motion. The young man not appearing for some time, and feeling responsible for his well-being, I sought him. The wound had been covered with soap and sugar, at the suggestion of a friend! On removing this, in place of before noted appearance, was now a broken, pulpy slough, instead of indurated integuments ; on displacing this, the tendons for two inches in length were disorganized and broken by slough, a few shreds of the under surface of one only remaining! The patient, though urged by myself and his employer, was unwilling to submit to treatment, and we retired, pained by anticipated result.

At intervals, meeting with the young man, we were permitted to note the progress and result—a union of the superficies by granulation and perfect restoration of the mobility of the fingers!

While upon the subject of tendons and their wounds, permit me to note a query for the purpose of asking a solution. The course of treatment, you are aware, suggested for ganglionic tumors or tendon-cyst is by pressure, rupture of the sac, puncture for discharge of contents and an endeavor to promote inflammation and adherence of the sides of the sac, absorption, by or excision. Each method has its advocates, and subject to modification or choice of the other. We have tried each of these methods and with varied success; the most certain of which is rupture, always excepting excision. This last we have practiced in preference to rupture, where counter-resistance to a blow did not promise well, by want of a bony-bed underlying, or through want of a tensely-filled sac.

Such a case presenting in the person of Mary M'K——, who had complained for past two years; the tumor (upon dorsum carpi) was accompanied with much pain, aggravated by necessary and almost constant use in sewing. Unable to give fixity to the tumor for rupture, or even pressure, its removal was determined, and that by a small crucial incision; by making this, speedily it was ascertained that the disease was not an 'unit,' but many; the incision was elongated to three inches, and on removal of two major, sundry minor ventricose tumors, size of a grain of rice and upwards to that of a bean, presented, inflated as it were from the outer covering of the tendons. In accordance with the popular requisition, these, and all appearance of sacs were removed, and the wound dressed for adhesion by first intention, which was happily induced. By result we had confirmation in this case that ruptures or any other mode could not by possibility have been successful.

In view of opinions entertained of necessity for utter extermination of sacs, and also of viability and recuperative property of tendons, the question presents, had these ventricose tumors been limited to one tendon solely, and that with the length of an inch or two, would it have been justifiable to remove by section this portion of diseased tendon? Other queries suggest themselves, as, what is this disease called ganglion? Is it allied to that which often presents at the wrist, and which Dr. Warren styles hydatid? The tumors above described did not have for contents other than the glairy fluid. W. T.

